# Mechanical Properties of CuZr Amorphous Metallic Nanofoam at Various Temperatures Investigated by Molecular Dynamics Simulation

**DOI:** 10.3390/ma18143423

**Published:** 2025-07-21

**Authors:** Yuhang Zhang, Hongjian Zhou, Xiuming Liu

**Affiliations:** 1 Hubei Digital Manufacturing Key Laboratory, School of Mechanical and Electronic Engineering, Wuhan University of Technology, Wuhan 430070, China; 2 School of Mechanical & Electrical Engineering, Wuhan Institute of Technology, Wuhan 430205, China; 3 Institute of Technological Sciences, Wuhan University, Wuhan 430072, China; liu_xiu_m@whu.edu.cn

**Keywords:** nanoporous materials, amorphous metals, mechanical properties, molecular dynamics, temperature effects

## Abstract

Metallic nanofoams with amorphous structures demonstrate exceptional properties and significant potential for diverse applications. However, their mechanical properties at different temperatures are still unclear. By using molecular dynamics simulation, this study investigates the mechanical responses of representative CuZr amorphous metallic nanofoam (AMNF) under uniaxial tension and compression at various temperatures. Our results reveal that the mechanical properties, such as Young’s modulus, yield stress, and maximum stress, exhibit notable temperature sensitivity and tension–compression asymmetry. Under tensile loading, the Young’s modulus, yield strength, and peak stress exhibit significant reductions of approximately 30.5%, 33.3%, and 32.9%, respectively, as the temperature increases from 100 K to 600 K. Similarly, under compressive loading, these mechanical properties experience even greater declines, with the Young’s modulus, yield strength, and peak stress decreasing by about 34.5%, 38.0%, and 41.7% over the same temperature range. The tension–compression asymmetry in yield strength is temperature independent. Interestingly, the tension–compression asymmetry in elastic modulus becomes more pronounced at elevated temperatures, which is attributed to the influence of surface energy effects. This phenomenon is further amplified by the increased disparity in surface-area-to-volume ratio variations between tensile and compressive loading at higher temperatures. Additionally, as the temperature rises, despite material softening, the structural resistance under large tensile strains improves due to delayed ligament degradation and more uniform deformation distribution, delaying global failure.

## 1. Introduction

Metallic nanofoams (MNFs) have drawn widespread interest because of their extraordinary characteristics, including extremely large surface area to solid volume ratio, lightweight structure, excellent catalytic activity, and outstanding mechanical performance [1,2,3,4,5,6]. Conventional MNFs with crystal lattices have been extensively fabricated, investigated, and applied. These materials demonstrate substantial potential in various applications, including flexible electronics, hydrogen energy storage/transportation, sensing, packaging, energy absorption, and chemical catalysis [7,8,9,10,11,12]. Understanding the fundamental mechanical behaviors and establishing the structure–property relationships of MNFs are essential for tailoring their performance and advancing their engineering applications. Substantial research has been dedicated to exploring the mechanical responses of MNFs with traditional crystalline metallic structures. For instance, Loaiza et al. [3] examined the strengthening mechanisms in MNFs like Cu, CuZn, and CuNi, finding that precipitation hardening significantly enhances the strength of MNFs, whereas solid solution strengthening has a less pronounced effect. Zhang et al. [13] devised a novel nanocomposite with a Cu substrate and a Cu MNFs surface layer using a liquid-assisted alloying–dealloying method. This nanocomposite exhibited superior mechanical properties under tensile loading, demonstrating an elongation no less than ~13.5%. The outstanding ductility can be ascribed to the superior interface adhesion between the Cu substrate and the Cu MNFs surface layer. [13]. Melis et al. [14] investigated the stiffening mechanisms in Au MNFs, discovering that local stress and strain fields around dislocation cores can serve as reinforcing agents, enhancing the stiffness of Au MNFs significantly.

Recently, amorphous alloys (AAs) have emerged as new parent materials for developing MNFs with bicontinuous porous structures [15,16,17]. For example, Fu et al. [16] fabricated a novel porous metallic glass using dissolvable templates, which is suitable for oil/water separation. The disordered arrangement of metallic elements in these alloys results in extraordinary mechanical performance, such as an elevated elastic limit, superior strength, and enhanced anti-wear properties. [18,19]. Amorphous metallic nanofoams (AMNFs) combine the benefits of AAs and MNFs and have been extensively developed and applied in various fields, including energy conversion [20], solar steam generation [21], and electrocatalysis [22]. Numerous studies have explored their mechanical behaviors under various loads. Pei et al. [20] introduced a NiP AMNF featuring hierarchical structures, which exhibited remarkable carbamide oxidation reactivity effectiveness owing to its high surface-to-volume ratio and distinctive chemical makeup. Zhang et al. [23] experimentally synthesized a ZrCuAl AMNF sample and systematically discussed its mechanical characteristics under compressive loading. Their findings revealed homogeneous deformation characteristics, contrasting sharply with the brittle fracture typically observed in bulk ZrCuAl glassy alloys [23]. The ZrCuAl metallic glass possesses an excellent strength-to-modulus ratio. Further insights were provided by Wang et al. [24], who examined the mechanical characteristics of ZrCuAl AMNF during tensile deformation. Their results indicated that the amorphous material effectively inhibits fracture initiation and suppresses crack propagation.

The Cu_50_Zr_50_ amorphous metallic material is a typical amorphous composition used to fabricate AMNF [23,24,25]. The mechanical properties of Cu_50_Zr_50_ AMNF under uniaxial tension have been studied [26,27,28]. However, the impact of temperature on the mechanical responses and underlying structural evolution of Cu_50_Zr_50_ AMNFs has been relatively underexplored. Temperature variations can significantly influence atomic mobility, surface energy dynamics, and surface stress distribution, leading to pronounced fluctuations in mechanical properties. A previous study [29] has shown that increased temperature can lead to lower stiffness and strength of AMNFs. However, the inherent atomistic events and deformation mechanisms under different temperatures are still unclear, leaving a gap in our knowledge of the temperature-dependent mechanical responses of AMNFs. How the surface stress and surface energy effects affect the mechanical behaviors of AMNFs at various temperatures remains unclear. Given that AMNFs exhibit nanoscale pores and solid ligament networks, revealing inherent nanoscale physical activities is crucial for comprehending their deformation phenomenon at various temperatures. Molecular dynamics (MD) simulation is a potent means to track these nanoscale deformation responses [30,31,32,33,34,35]. For example, Yang et al. [30] revealed the deformation responses of porous metallic glass under tensile and cyclic loading. To address these gaps in the temperature-dependent mechanical behaviors of AMNFs, this work uses MD simulations to systematically investigate the temperature-dependent deformation mechanisms and mechanical features of AMNFs. How the surface state, including surface energy and surface stress, affects the mechanical properties and deformation modes is the focus of the paper. In this regard, we aim to elucidate the underlying mechanisms that drive the observed tension–compression asymmetry and the temperature-dependent mechanical behaviors of AMNFs. Our findings are expected to advance the fundamental comprehension of the mechanical behaviors of AMNFs at different working temperatures and provide valuable insights for the development and optimization of nanostructured metallic glasses with tailored properties for specific applications.

## 2. Models and Methods

**Figure 1:**
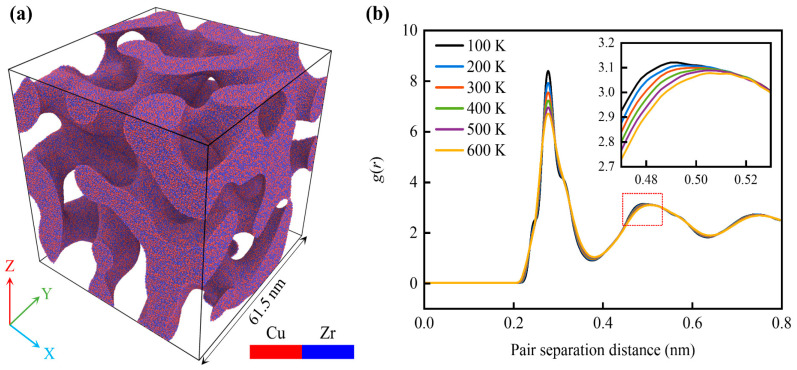
(**a**) Atomistic visualization of the Cu_50_Zr_50_ AMNF architecture, depicting the complete three-dimensional atomic arrangement. (**b**) Computed radial distribution profiles of atomic pair correlations for the fully relaxed AMNF system at different temperatures.

In the current work, to reveal the temperature sensitivity of nanoscale deformation events and mechanical properties of CuZr AMNF, MD simulations are executed leveraging the publicly available Large-scale Atomic/Molecular Massively Parallel Simulator (LAMMPS) computational toolkit, specifically designed for large-scale atomistic modeling [36]. The visualized analysis and post-processing of atomistic results are performed using the Open Visualization Tool (OVITO) [37]. The extensively used embedded atom method (EAM) potential introduced by Cheng et al. [38,39,40] for CuZr AA is adopted. Constructing a realistic nanoscale model of AMNF with pore and ligament structures identical to the specimen fabricated via dealloying corrosion in experiments is crucial for precise atomistic simulations of mechanical tests. For detailed insights into the fabrication processes of random bicontinuous AMNF, readers are directed to prior related studies [41,42,43]. Briefly, bulk Cu_50_Zr_50_ AA precursor, characterized by a disordered atomic distribution, is synthesized using the classical melt-quenching method [32,44,45]. The bulk Cu_50_Zr_50_ AA is used as the bulk precursor to construct the AMNF with a bicontinuous void-solid microstructure. Similar to previous works [46,47,48], the spinodal evolution described by the classical Cahn–Hilliard equation [49] can be simulated to establish the bicontinuous porous nanomaterial:

(1) $$\frac{\partial u}{\partial t}=\nabla^{2}\left[\frac{df(u)}{du}-\theta^{2}\nabla^{2}u\right]$$

where ∇ is the gradient operator; *u* is the concentration difference of the two phases, and it is a function of space positions (*x*, *y*, *z*) and system evolution time *t*; *f*(*u*) is the free energy function; and θ is the transition region width between the two phases. In this paper, $f(u)={\left(u^{2}-1\right)}^{2}/4$ and θ=0.01 are used according to the previous reference [50]. By tuning parameters during the spinodal decomposition process (critical concentration difference and system evolution time), structural features of the AMNF, such as average ligament size (thickness) and solid fraction, can be precisely controlled. Hence, the structure–property relationship of the AMNF can be studied. As shown in Figure 1a, the fabricated AMNF atomistic model is enclosed in a cubic box with a side length of 61.5 nm, a relative density (*ρ*) of 0.40, and an average ligament size (*d*) of approximately 10 nm. The characteristic dimensions of the ligament network, specifically referring to the mean cross-sectional diameter of the individual ligaments, are quantitatively determined through computational image processing utilizing the specialized software AQUAMI (written in Python version 3.6) [51]. Figure 1a also reveals that Cu and Zr atoms are uniformly and randomly distributed within the solid ligament network, confirming the disordered atomic configuration. A total of ~5.4 million atoms are included in the sample. Note that only one sample is established and subjected to mechanical tests at different temperatures to explore the temperature-dependent mechanical behaviors.

To eliminate initial instabilities in the AMNF atomic model, energy minimization is performed by resorting to the conjugate gradient (CG) algorithm, followed by a 100 ps relaxation period at the target temperature and ambient hydrostatic pressure of 0 GPa under the NPT (isothermal-isobaric) ensemble. All simulations are conducted at environmental temperatures varying from 100 K to 600 K in increments of 100 K. Periodic boundary conditions (PBCs) are implemented along all three Cartesian axes to mitigate boundary effects and simulate a larger atomistic system. A timestep of 0.002 ps is employed. After achieving sufficient equilibrium, MD simulations are conducted to investigate the temperature-dependent mechanical properties of the AMNF. The temperature control in our MD simulations is implemented through the Nosé–Hoover thermostat. The simulated mechanical testing protocol involves uniaxial deformation along the Z-coordinate at a prescribed strain rate of 5 × 10^8^ s^−1^, encompassing both tensile and compressive modes. The stress state in transverse directions is regulated by employing the NPT thermodynamic ensemble, which maintains atmospheric pressure conditions (0 GPa) along both X- and Y-axes throughout the computational tests. This modeling and mechanical performance simulation method for nanoporous metallic materials has been extensively employed and validated by numerous studies, ensuring its accuracy and reliability [52,53,54]. The structural analysis depicted in Figure 1b illustrates the temperature-dependent radial distribution function *g*(*r*) for the completely relaxed AMNF configuration. The observed splitting and broadening of the second coordination sphere in the pair correlation function serve as distinctive signatures of the non-crystalline atomic arrangement, confirming the amorphous phase stability under varying thermal conditions. The height of the first peak grows as the temperature decreases, indicating greater structural stability at lower temperatures. In addition, the position of the second peak shifts to the right with rising temperature. This implies that the atomic distance becomes larger as the temperature rises.

## 3. Results and Discussion

### 3.1. Stress–Strain Responses

**Figure 2:**
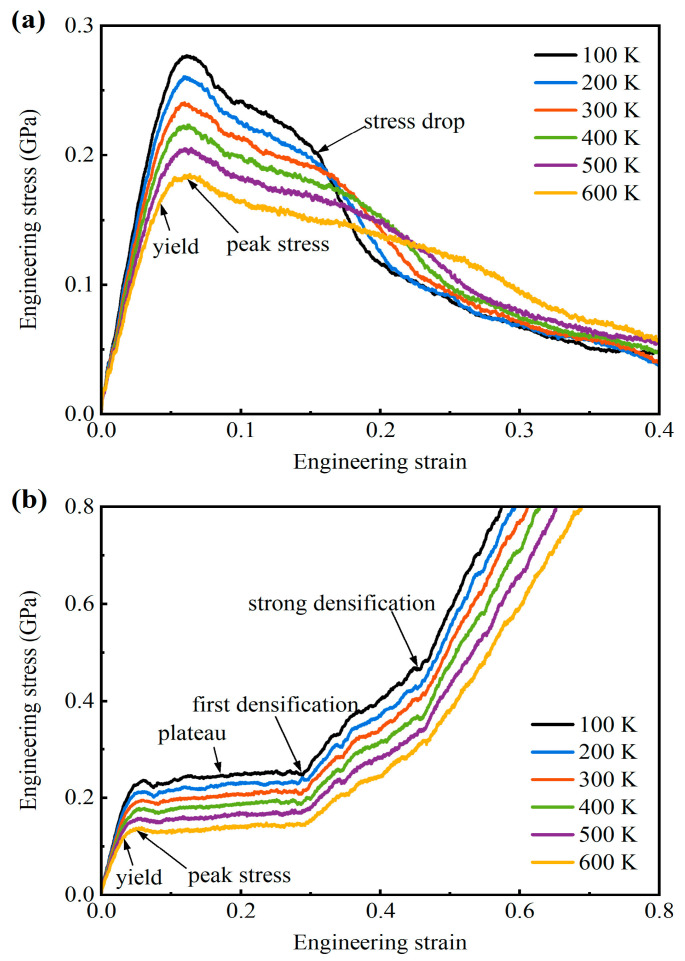
(**a**) Uniaxial tensile and (**b**) compressive engineering stress–strain behaviors of the simulated AMNF specimen at different temperatures varying from 100 to 600 K with an interval of 100 K.

Figure 2 presents the obtained uniaxial tensile and compressive global stress versus strain graphs for the tested AMNF sample at various environmental temperatures. Under tensile loading, as Figure 2a illustrates, the engineering stress rapidly increases to a small percentage of engineering strain in a linear path, suggesting the initial linear elastic deformation event of the AMNF. Following the elastic limit, the stress continues to increase with strain briefly, exhibiting slight strain-hardening behavior. This strain-hardening behavior is because of the change in deformation behavior from network bending to network tension. Following the achievement of peak engineering stress in the stress-strain response, the AMNF yields, and the global engineering stress gradually reduces due to the ligament decay, indicating the diminishing resistance capability of the porous structure. Therefore, the deformation phenomenon of the AMNF under uniaxial tensile loading is divided into three regions: (1) initial linear elastic deformation region, (2) strain-hardening region after yielding, and (3) ligament decay region. It can be noted that during the ligament decay region, the engineering stress initially decreases with strain at a relatively low rate. This smooth decrease in engineering stress occurs because of ligament necking and softening due to excess plastic deformation. After a certain plastic strain, a remarkable stress drop emerges, as indicated in Figure 2a. Subsequently, global stress sharply declines as the strain rises. The underlying mechanism behind this rapid decrease in stress is because of the fracture of numerous ligaments. Following this stress drop regime, the stress continues to reduce with strain at a milder rate. Notably, this characteristic of stress drop is more distinct for samples at lower temperatures. Specifically, at a relatively smaller temperature of 100 K, the point at which the stress drop begins can be clearly identified. However, at the highest temperature of 600 K, the stress drop is less pronounced. In addition, one can note that during the elastic response and the early ligament decay stages, the engineering stress at a specific strain point is larger for samples with higher environment temperatures. This is the common high-temperature-softening behavior observed in engineering materials. However, at larger plastic strains (larger than ~0.3), the stress of the sample at higher temperatures is even higher than that at lower temperatures.

As Figure 2b shows, under uniaxial compressive loading, the AMNF exhibits linear elastic response, transient strain-hardening, plateau stress flow, and densification responses. Interestingly, densification is achieved in two steps: (1) first densification, where the stress-strain curve departs the plateau stage and the engineering stress remarkably enhances with strain; (2) strong densification, where the stress significantly rises with strain at much higher rates than the first densification. At the same global compressive strain, the stress is greater for samples tested at higher temperatures. The plateau deformation is critical for the energy absorption of porous materials. It is observed that the strain range of the plateau stage is insensitive to temperature, but the plateau stress is higher at lower temperatures. Therefore, the AMNF exhibits superior energy absorption efficiency in the plateau stage at lower temperatures.

### 3.2. Mechanical Parameters at Different Temperatures

The mechanical parameters of the AMNF across various simulated temperatures are obtained, including the tensile/compressive Young’s modulus *E*, yield stress *σ*_y_, maximum stress *σ*_p_, and yield strain *ε*_y_. The Young’s modulus *E* of the AMNF is calculated using the engineering stress–strain data up to the engineering strain scope of 0 to 0.025, where the stress exhibits a linear relationship with the engineering strain. The yield strength *σ*_y_ and the yield strain *ε*_y_ are obtained by resorting to the strain offset method with a 0.002 plastic strain. The maximum stress for tensile loading is defined as the maximum value of engineering stress during the tensile deformation cycle, and the maximum stress for compressive loading is defined as the first peak value of engineering stress, as indicated in Figure 2b.

**Figure 3:**
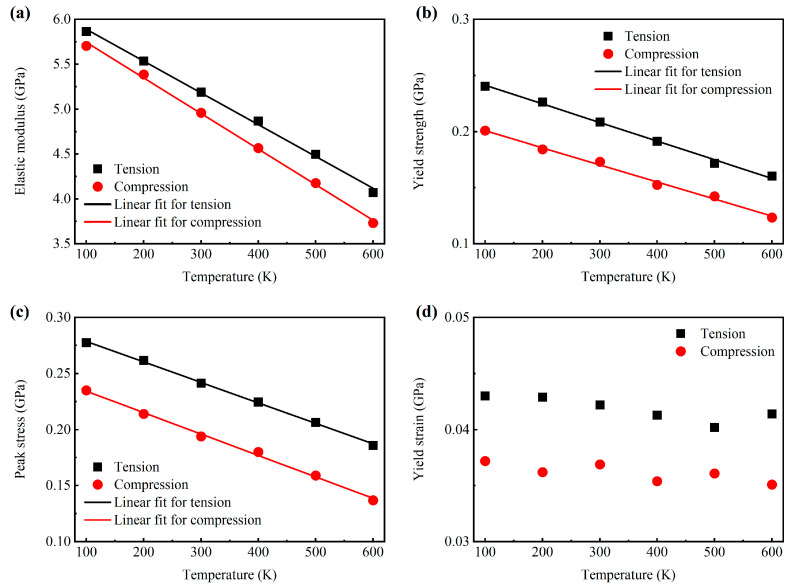
Variation of the mechanical parameters of the AMNF under tension and compression with simulated temperature: (**a**) Young’s modulus, (**b**) yield strength, (**c**) peak stress, and (**d**) yield strain. The black square point and the red circle point correspond to the mechanical properties under tensile loading and compressive loading, respectively.

Figure 3 presents the Young’s modulus, yield stress, peak stress, and yield strain of the AMNF as a function of the environmental temperature. Distinctly, the Young’s modulus, yield strength, and peak stress elevate with the drop in temperature. After fitting data for these mechanical parameters as a function of temperature, it is found that the three mechanical parameters are linearly related to temperature. The coefficient of determination *R*^2^ is greater than 0.99, indicating that the linear fits are highly accurate.

These temperature-dependent linear trends in mechanical features are fundamentally governed by the amplified thermal agitation of atomic constituents at higher temperatures. The enhanced kinetic energy of atoms results in decreased potential barrier heights and modified interatomic interactions, thereby influencing the macroscopic mechanical properties. As the temperature increases, the interatomic distance expands, leading to a reduction in binding energy between atoms [55,56]. Consequently, the material undergoes softening, which facilitates atomic displacement and results in a decrease in flow stress during tensile testing. Furthermore, Young’s modulus serves as a direct indicator of the bond energy between atoms within a material. Thus, at lower temperatures, where atomic bond energy is higher, materials exhibit a corresponding increase in Young’s modulus. However, as Figure 3d represents, the yield strain does not exhibit a clear trend with the increase in temperature, and it varies minimally with temperature. Another thing that should be highlighted is that the Young’s modulus, yield strength, peak stress, and yield strain under tension are greater than the corresponding parameters under compression. This suggests that the AMNF manifests intense tension–compression asymmetry. This phenomenon originates from the combined effects of nanoscale surface energetics and intrinsic surface stress characteristics, which will be quantitatively examined in Section 3.5.

The obtained values of the elastic modulus under tension (*E*_t_) and compression (*E*_c_) at different simulated temperatures are tabulated in Table 1. The tensile Young’s modulus of the AMNF at 100 K is 5.863 GPa. Previous work by Liu et al. [26] established that AMNF structures with a relative density of 0.40 and an average ligament diameter of ~4 nm possess a Young’s modulus of 5−6 GPa under tensile loading at cryogenic temperature (50 K). The quantitative discrepancy between our measured values and the literature data can be reasonably explained by considering the combined effects of strain rate sensitivity and variations in ligament morphology. Zhang et al. [23] and Wang et al. [24] experimentally measured the mechanical properties of AMNF. However, the chemical compositions, ligament sizes, and relative densities of the structures tested by them [23,24] are not comparable to those in this work. As Table 1 shows, through the thermal progression from cryogenic (100 K) to elevated temperatures (600 K), the elastic modulus under tension and compression decreases by approximately 30.5% and 34.6%, respectively. The results demonstrate a notable and critical temperature sensitivity of Young’s modulus of the AMNF, which increases with dropping temperature. This trend is expected because the change in the yield strain among different temperatures is slight, while that in the yield strength is considerably more pronounced. The asymmetric response in elastic properties under tension and compression (*E*_t_ − *E*_c_) is calculated and listed in Table 1 to reflect the extent of the tension–compression asymmetry of elastic modulus. One can note that, except in the case at 100 K, the value of *E*_t_ − *E*_c_ increases with increasing temperature. This implies that the tension–compression asymmetry of modulus becomes more dramatic at higher temperatures. Unfortunately, the existing related literature focusing on reported experimental values of the Young’s modulus asymmetry at different temperatures is lacking, preventing comparison with our findings. The mechanical anisotropy between tensile and compressive responses is principally governed by surface effects, particularly surface stress and surface energy contributions. These nanoscale phenomena exhibit strong dependences on the surface-area-to-volume ratio, which dictates the relative influence of surface atoms versus bulk material. As the temperature changes, the amplitude of the atomic fluctuations and the global thermal dilation change, resulting in variations in the surface properties. In the later subsection, the inherent mechanisms behind this temperature effect of tension–compression asymmetry will be discussed in detail. Saffarini et al. [57] demonstrated that the discrepancy between the tensile elastic modulus and compressive elastic modulus of traditional Au MNFs is ~1.72 GPa and does not change with temperature. The difference for AMNF increases from 0.150 to 0.339 GPa, with the temperature increase from 200 to 600 K, as shown in the last column in Table 1. This suggests that the tension–compression asymmetry is less pronounced, but its temperature sensitivity is stronger for AMNF compared to traditional Au MNFs.

The tensile/compressive yield strength (*σ*_yt_ and *σ*_yc_) and tensile/compressive yield strain (*ε*_yt_ and *ε*_yc_) of the AMNF determined using the offset method at various temperatures are summarized in Table 2. At ambient temperature conditions (300 K), the AMNF exhibits distinct yield strengths of 0.209 GPa under tensile loading and 0.173 GPa when subjected to compressive deformation, demonstrating a notable tension–compression asymmetry. Experimentally, tensile and compressive yield strength values of 0.22−0.23 GPa have been reported for AMNF at room temperature with a solid fraction (relative density) of 0.509 and an average pore wall size of 20.84 nm [23]. The data in Table 2 confirm the strong dependence of the yield stress of the AMNF on environmental temperature. Specifically, the tensile and compressive yield strengths increase by ~50% and 62%, respectively, as the temperature reduces from 600 to 100 K. However, the tension–compression asymmetry for yield strength (*σ*_yt_ − *σ*_yc_) shows no significant temperature dependence, as shown in the last column in Table 2. The tensile yield stress is distinctly higher than that under compression. This phenomenon, consistently observed in various MNFs through both computational modeling and experimental characterization [58,59], originates from surface stress effects. A comprehensive analysis of this tension–compression asymmetry mechanism will be presented in Section 3.5, focusing on the surface-dominated yielding behavior. Additionally, Table 2 shows that the yield strain and its tension–compression asymmetry do not exhibit a specific trend with temperature variation, and exhibit very mild temperature sensitivity.

**Figure 4:**
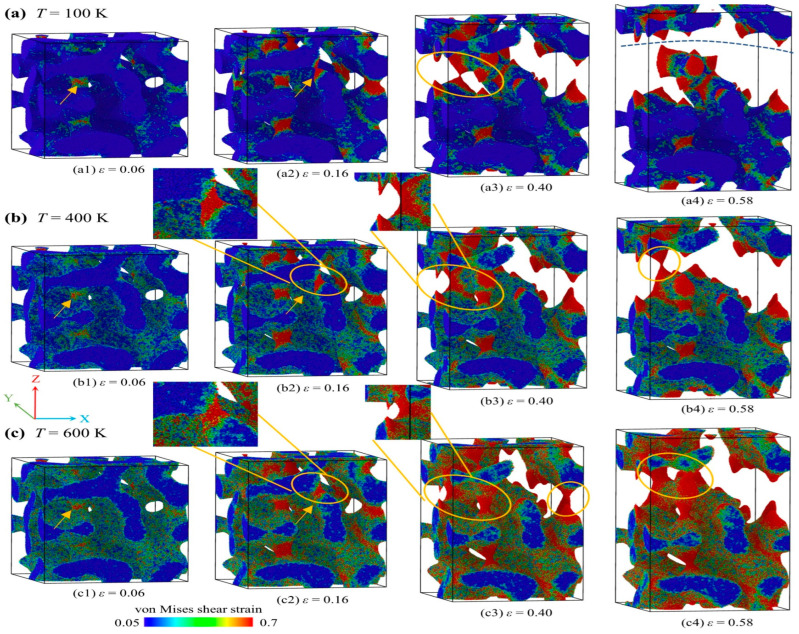
Uniaxial tensile deformation processes of the AMNF at different temperatures of *T* = (**a**) 100 K, (**b**) 400 K, and (**c**) 600 K. Atoms are colored by their local shear strain values to represent the distribution of local strain.

**Figure 5:**
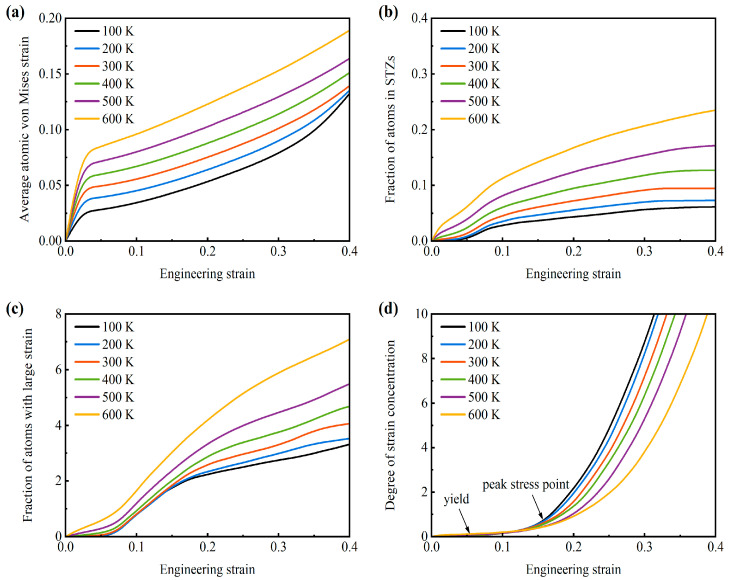
Change of (**a**) average atomic von Mises shear strain, (**b**) fraction of atoms in STZs, (**c**) extent of deformation localization, and (**d**) percentage of atoms having shear strain greater than 0.7 with global strain for the AMNF sample under tensile loading at different temperatures.

Table 3 summarizes the peak stress (*σ*_pt_) and the stress and strain values at the start point of the stress drop for the AMNF under tension at different temperatures. The tensile peak stress at 300 K is 0.242 GPa. Experimentally, tensile peak stress of ~0.250 GPa has been reported for AMNF with a porosity of 0.47 and a pore size of 10.66 nm [24]. As the temperature rises, the tensile peak stress of the AMNF remarkably decreases. Nonetheless, the ratio of peak stress to yield strength (*σ*_pt/_*σ*_yt_) remains relatively unaffected by temperature. The peak stress is higher than the yield strength within the range of 15−20%. For Au MNFs [57], the tensile peak stress can exceed the yield strength by ~5%. This observation clearly demonstrates that the strain-hardening capacity of AMNF substantially surpasses that of their gold-based MNF counterparts. The results in Table 3 demonstrate that the stress value at the stress drop point is smaller for samples at higher temperatures, while the strain at the stress drop point is the opposite. At the stress drop point, the engineering stress sharply declines, significantly reducing the mechanical resistance of the material. Therefore, the ductility of the structure can be characterized by the strain at the onset of the stress drop. Table 3 demonstrates that the strain at the stress drop point increases with rising temperature. This confirms that the ductility of the AMNF under tension is improved as temperature increases. At higher temperatures, the thermal fluctuation of atoms is more intense, and atoms tend to deviate more easily from their equilibrium positions. As a result, more atoms are engaged in the deformation process (Figure 4 and Figure 5). The global and homogeneous deformation is promoted, and the degree of localized deformation is reduced, enhancing the ductility of the material (Figure 4 and Figure 5).

### 3.3. Deformation Modes Under Tension

In this subsection, we elucidate the deformation modes of the AMNF under tensile loading across various temperatures. The plastic deformation behaviors of typical crystalline MNFs include dislocation slip, twinning, phase transformation, grain boundary migration, and formation of stacking faults. However, such crystalline defects are inherently lacking in AAs due to their unique disordered atomic packing structure. It is well known that the nucleation and progression of local shear transformation zones (STZs) [32,60] and the activities of shear bands are the major carriers of plastic deformation in AAs.

To elucidate the intrinsic plastic deformation mechanisms of the AMNF, the von Mises shear strain [61] of each atom within the atomistic model is monitored. Figure 4 presents the atomic shear distribution of the AMNF at four distinct global tensile strain levels: *ε* = 0.06, 0.16, 0.40, and 0.58, under temperatures of 100 K, 400 K, and 600 K. The atomic configurations shown in Figure 4 are generated from MD simulations and analyzed using OVITO [37] to visualize local strain distribution. Note that the atoms are color-coded based on their respective von Mises shear strain values. It can be seen that the change in temperature does not result in qualitative alteration in the shear strain distribution, but it does have quantitative effects. During the initial elastic deformation regime, the underlying deformation mode of the AMNF involves the rotation/bending and stretching of the solid amorphous ligaments [29]. Following global yielding, plastic strain initiates and evolves, revealing a fascinating intrinsic deformation mechanism. In crystalline MNFs, crack formation is more prevalent compared to metallic nanowires, primarily due to their intricate geometric microstructures. The ligaments within MNFs exhibit random and irregular morphologies, including variations in shape, size, connectivity, and surface curvature. As a result, when subjected to external loads, the weakest regions, i.e., typically the thinnest networks or areas with stress concentration, are prone to fracture first, initiating crack formation. These cracks then propagate sequentially through the network via necking and fracture/separation of individual ligaments, ultimately leading to the complete failure of the MNFs.

As shown in Figure 4(a1–c1), at *ε* = 0.06, the stress reaches its approximate maximum. Localized intense plastic strains emerge in stress concentration regions and the narrower sections of the ligaments, as indicated by the orange arrows in Figure 4(a1–c1). This severe plastic strain leads to the degradation of the solid ligament skeletons. Once the maximum tensile stress is attained, the stress exhibits continuous attenuation. The underlying plastic deformation mechanism involves progressive necking and ductile fracture of the solid ligaments. Temperature exerts a significant influence on the tensile deformation behavior of the AMNF. As temperature increases, both the number of atoms participating in plastic deformation and the extent of global homogeneous deformation rise. The area and intensity of plastic deformation also expand with increasing temperature. Specifically, as illustrated in Figure 4a, at 100 K, pronounced plastic deformation occurs locally in the necking and fracture regions of the ligaments, while other regions exhibit only mild deformation, with almost no green or yellow atoms observed. Plastic deformation is primarily confined to the ligament surfaces, with minimal activity in the core regions. In contrast, at the higher temperature of 600 K, plastic deformation extends beyond stress concentration zones to other regions. Nearly all atoms on the ligament surfaces undergo substantial plastic strain (evidenced by yellow and green atoms), and shear transformation zones (STZs) propagate from the ligament surfaces into the cores. Another key effect of temperature is its modification of ligament necking and fracture behavior. As indicated by the orange arrow in Figure 4(a2), at *ε* = 0.16, intense strain localization causes the weakest ligament to undergo severe necking, nearing fracture. However, at elevated temperatures (400 K and 600 K), necking in the same ligament is less pronounced, as shown in Figure 4(b2,c2). At a larger strain (*ε* = 0.40), multiple ligaments exhibit necking and complete fracture at 100 K (Figure 4(a3)), whereas at 400 K and 600 K, the number of fractured ligaments decreases significantly. By *ε* = 0.58, a major crack fully propagates through the AMNF at 100 K (dotted line in Figure 4(a4)), resulting in structural failure. At 400 K, one ligament remains intact (orange circle in Figure 4(b4)), while at 600 K, necking and rupture are minimal. In summary, higher temperatures promote greater atomic participation in plastic deformation and reduce strain localization. This delays ligament necking and fracture, enhancing the ductility of the AMNF. Consequently, at large plastic strains (beyond *ε* = ~0.3), higher temperatures yield greater engineering stress compared to lower temperatures, as seen in Figure 2a.

The evolution of statistical characteristics of atomic von Mises shear strain for the AMNF under tensile loading is calculated and discussed quantitatively. Figure 5a demonstrates the average local atomic strain of all atoms inside the atomistic specimen under tension as a function of global engineering strain across various temperatures. It can be seen that the average von Mises shear strain increases monotonically with the increase in engineering strain. At a given strain point, the average von Mises shear strain is larger for samples at higher temperatures. This means that the global deformation degree has a positive relationship with the temperature, similar to the intuitive observation in Figure 4. Figure 5b plots the change in the percentage of atoms in STZs with engineering strain for different temperatures. The atoms with von Mises shear strain larger than 0.2 but smaller than 0.7 are considered as in the STZs. The curve in Figure 5b illustrates that the fraction of atoms in STZs remarkably enhances with increasing strain and increasing temperature. This phenomenon confirms that more atoms engage in a homogeneous plastic flow state at higher temperatures. At relatively higher temperatures (300–600 K), once the tensile strain is imposed, the fraction of atoms in STZs rises, which indicates the generation of STZs. However, at relatively lower temperatures (100−200 K), the fraction of atoms in STZs remains zero during the initial few percent of strain. Figure 5c reflects the variation of atoms with a local strain larger than 0.7. These atoms have larger shear strain values and are detrimental to the strength of the material. One can note that at the same strain point, the percentage of atoms with large shear strain rises as the temperature elevates. As a result, the strengths (yield strength and ultimate tensile strength) of the AMNF drop with rising temperature. The degree of strain concentration ψ during the deformation process is defined as:

(2) $$\psi =\sqrt{\frac{1}{N}\sum_{i}^{N}{\left(\eta_{\mathrm{Mises}}^{i}-\eta_{\mathrm{Mises}}^{\mathrm{ave}}\right)}^{2}}$$

where *N* is the total number of atoms inside the atomistic model, $\eta_{\mathrm{Mises}}^{i}$ is the local von Mises shear strain of the *i*-th atom, and $\eta_{\mathrm{Mises}}^{\mathrm{ave}}$ is the average value of the von Mises shear strain of all atoms. In essence, ψ is the standard deviation of the per-atom von Mises shear strain and is a dimensionless quantity. A large value of ψ indicates that the sample has been severely locally deformed. A small value of ψ implies homogeneous global deformation. As shown in Figure 5d, the degree of deformation localization smoothly rises with increasing strain just after global yielding. Subsequently, after the peak stress is reached, the deformation concentration degree increases with strain at a much higher rate. This variation tendency suggests that the deformation is relatively uniform and homogeneous before reaching the maximum tensile stress. However, during the stress decay stage, the deformation mode is localized due to the ligament necking and breakage. Another thing that can be noted from Figure 5d is that the degree of strain concentration is more intense for samples at lower temperatures. This increased localization deformation mode at lower temperatures causes more distinct and earlier stress drops at the stress decay stages, as shown in Figure 2a and Table 3.

**Figure 6:**
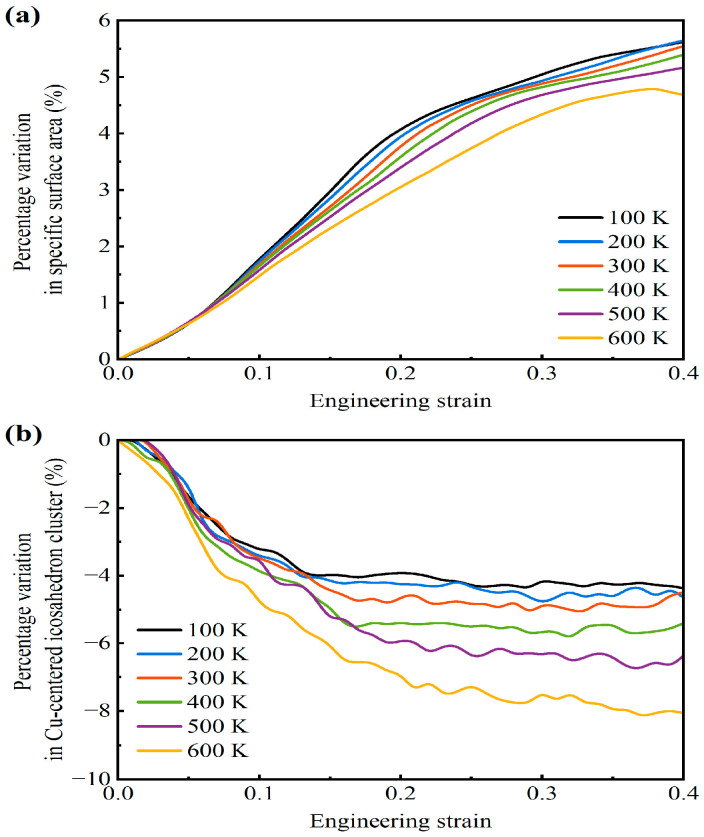
(**a**) Percentage variation in surface-area-to-solid-volume ratio, and (**b**) percentage variation in the number of Cu-centered icosahedron clusters with global strain for the AMNF sample under uniaxial tension.

Figure 6a describes the percentage change in the surface-area-to-solid-volume ratio of the AMNF sample under tensile loading at various temperatures. As the engineering strain rises, the surface-area-to-solid-volume ratio increases steadily. The increasing surface-area-to-solid-volume ratio is because of the necking and rupture of ligaments, which form new free surfaces. At higher temperatures, the increasing rate of the percentage variation in the surface-area-to-solid-volume ratio slows markedly. Therefore, it can be concluded that the necking and rupture of ligaments are postponed as temperature increases. This quantitative result is similar to the direct observation in Figure 4.

It has been widely reported that the atomic structures of AAs can be comprehended from the view of Cu-centered clusters. The cluster can be indicated by a Voronoi polyhedron with the index <*n*_3_, *n*_4_, *n*_5_, *n*_6_>. The Cu-centered Voronoi icosahedral cluster <0, 0, 12, 0> and networks are crucial building blocks for AA materials, and they significantly contribute to the mechanical performance of AAs. Therefore, the percentage variation in the population of the Cu-centered Voronoi icosahedral cluster <0, 0, 12, 0> is tracked for the tested AMNF, as plotted in Figure 6b. The population of the Cu-centered Voronoi icosahedral cluster <0, 0, 12, 0> initially decreases steeply with the engineering strain. This indicates that the Cu-centered icosahedral cluster collapses and disintegrates. After a certain engineering strain, the decreasing rate in the Cu-centered icosahedral cluster markedly slows. This is because the activity and development of STZs are essentially completed. The decrease in icosahedral clusters at higher temperatures is evidently steeper than that at lower temperatures. Therefore, the enhancement in temperature might cause more severe internal damage to the material properties. From the perspective of material properties, the enhancement in temperature causes material softening, leading to lower stiffness and strength of the solid ligaments. However, from the perspective of porous structure, the delay of ligament necking and rupture at higher temperatures improves the ductility and response stress at relatively large strains (larger than ~0.3).

### 3.4. Deformation Modes Under Compression

**Figure 7:**
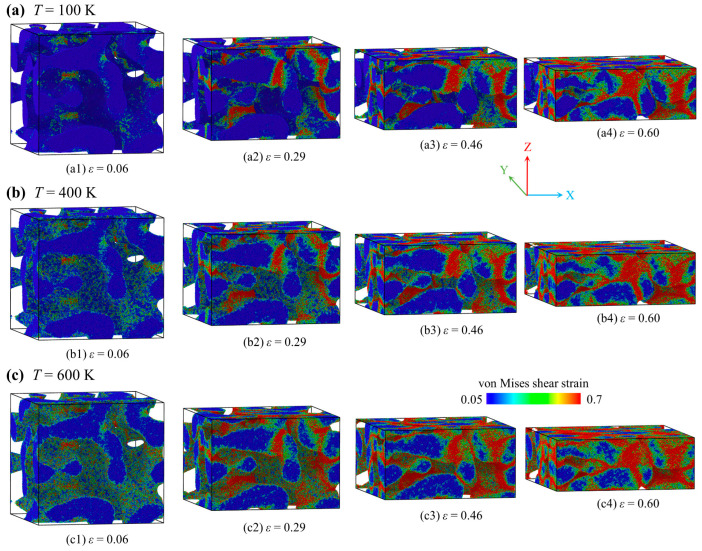
Uniaxial compressive deformation processes of the AMNF at different temperatures of *T* = (**a**) 100 K, (**b**) 400 K, and (**c**) 600 K. Atoms are colored by their local strain values to represent the distribution of local strain.

Having elucidated the nanoscale tensile deformation mechanisms of the AMNF, we now examine its compressive behavior at temperatures of 100 K, 400 K, and 600 K. Figure 7 illustrates the deformation evolution at global compressive strains of *ε* = 0.06, 0.29, 0.46, and 0.60, with atoms color-coded by their local atomic shear strain. Under compressive loading, the AMNF initially demonstrates a linear elastic response, followed by yielding at small strains and a transition to elastic-plastic deformation. At *ε* = ~0.06, pronounced strain localization emerges (Figure 7(a1–c1)), coinciding with a drop in global stress and the onset of a plateau regime, where stress stabilizes. During this stage, localized plastic deformation expands significantly, particularly in regions undergoing geometric distortion and along ligament surfaces due to the mutual extrusion of skeletal structures. At higher strains (*ε* = 0.29 and 0.46), densification dominates as ligaments contact and coalesce, forming a unified structure. While temperature does not alter the fundamental deformation modes, it quantitatively affects atomic shear strain distribution. As shown in Figure 7a, elevated temperatures engage more atoms in compressive deformation, especially within ligament surface layers, promoting more homogeneous strain accommodation.

**Figure 8:**
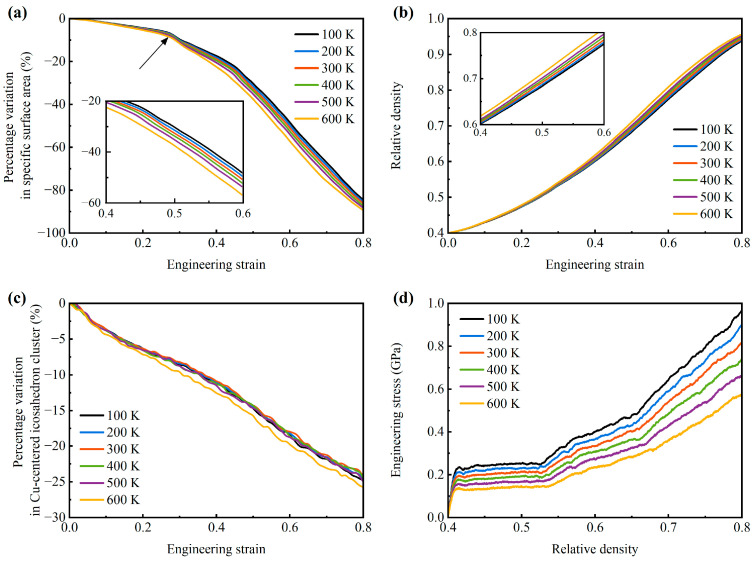
(**a**) Percentage variation in surface-area-to-solid-volume ratio, (**b**) relative density, and (**c**) percentage variation in the number of Cu-centered icosahedron clusters with engineering strain for the AMNF sample under uniaxial compression. (**d**) Engineering stress as a function of relative density for the AMNF sample under uniaxial compression.

To quantitatively understand the strain-induced densification behavior for the AMNF under compressive loading, Figure 8a and Figure 8b reflect the percentage variation in surface-area-to-solid-volume ratio and relative density with compressive engineering strain for the AMNF, respectively. One can observe that once the compressive strain is applied, the surface-area-to-solid-volume ratio reduces, and the solid fraction of the porous material increases with global strain. This implies that the densification is induced from the beginning of compressive loading and is continuously reinforced during the deformation process. As the black arrows in Figure 8a denote, the surface-area-to-solid-volume ratio first decreases mildly with strain. After a certain strain, it reduces with strain at relatively steeper rates. The transition strain for the decreasing rate of the surface-area-to-solid-volume ratio is about ~0.27−0.29, corresponding to the first densification point in Figure 2. After the first densification, the variation in the decreasing rate of the surface-area-to-solid-volume ratio is very slight. Hence, the first densification is much stronger than the second densification. As the temperature increases, the decreasing rate of the surface-area-to-solid-volume ratio and the increasing rate of the relative density are enhanced. This means the densification phenomenon is more intense at higher temperatures. The underlying reason is that the atomic distance and potential energy of atoms are greater at higher temperatures. Hence, the ligament surfaces more easily come into contact with each other and merge into one solid. Nonetheless, it can be seen from Figure 8a,b that the effects of temperature on the densification degree of the AMNF are mild. Figure 8c presents the percentage variation in the population of the Cu-centered Voronoi icosahedral cluster <0, 0, 12, 0> with compressive engineering strain. With the increasing strain, the population of the Cu-centered icosahedral decreases, indicating the damage caused by high stress. It can be noted that the effect of temperature on the disintegration of the Cu-centered icosahedral is very mild. Figure 8d displays the engineering stress-relative density relationship for the AMNF under compression. Observe that with the same relative density, the engineering stress increases with dropping temperature. A larger engineering stress is required to meet the same relative density for the AMNF at lower temperatures.

**Figure 9:**
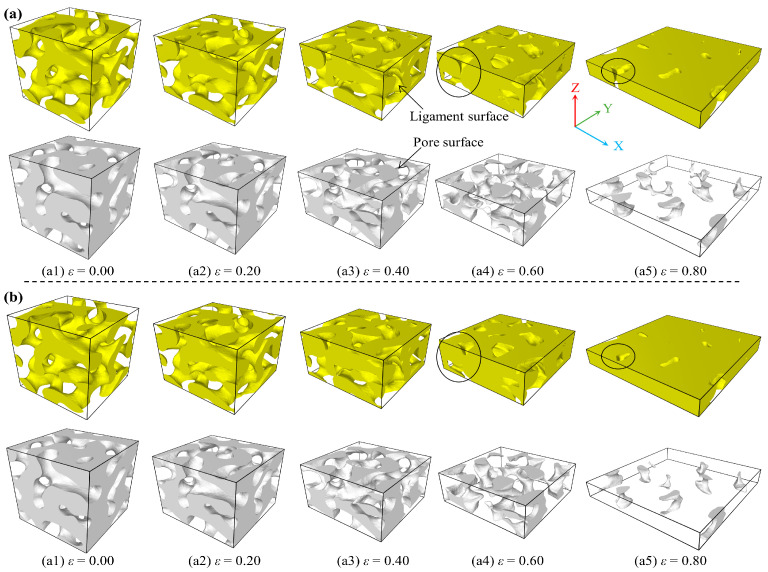
3D deformation pattern for the AMNF during compression deformation at specific engineering strains with an interval of 0.20 under different temperatures of (**a**) 100 K and (**b**) 600 K. The ligament surface and void surface are colored yellow and grey, respectively.

To intuitively comprehend the densification and void collapse behaviors of the AMNF, Figure 9 represents the solid ligament network surface (colored yellow) and the void network surface (colored grey) change for the AMNF at various compressive engineering strains. The solid and void network surfaces are identified by OVITO [37] with a probe radius of 0.3 nm. One can clearly observe that during the deformation regime, the ligament networks are compressed and come in contact with each other. When the ligament surfaces are self-touching, one integral is generated. The voids collapse and are annihilated as a result of the coalescence of ligaments. Note that the initial microstructures of the AMNF are stochastic and continuous, i.e., the void and solid networks are extended throughout the 3D space. Interestingly, after a certain engineering strain, the voids are no longer distributed continuously because of the collapse of voids and the merging of ligaments. The network of void surfaces gradually fades away and transforms into discrete voids. The AMNF undergoes a gradual transformation from an open-cell porous configuration to a closed-cell structure. This closed-cell material can be conceptualized as a bulk material interspersed with isolated voids. Even at the maximum engineering compressive strain of 0.80, the pores are not entirely eliminated, and some voids remain observable, as shown in Figure 9(a5,b5). The variation of the solid and pore networks, depicted in Figure 9, highlights subtle discrepancies in the densification behaviors of specimens at varying temperatures. Specifically, the densification is more intense at higher temperatures. In other words, the collapse and annihilation of pores are delayed at lower temperatures. As indicated by the black circles in Figure 9, it is evident that the residual volume and number of voids are larger for the sample at a lower temperature.

### 3.5. Tension–Compression Asymmetry

**Figure 10:**
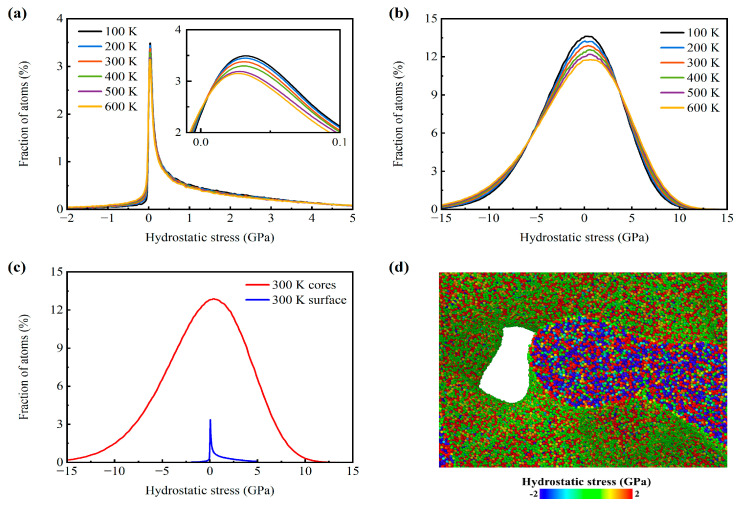
Initial per-atom stress state for the AMNF after adequate relaxation at various temperatures before loading. Atomic hydrostatic stress distribution of atoms in (**a**) ligament surface layers and (**b**) ligament cores for the sample at different temperatures. (**c**) Comparison of the atomic hydrostatic stress distribution between the surface atoms and interior atoms at 300 K. (**d**) Partially enlarged view of one ligament inside the sample at 300 K, with atoms colored according to their hydrostatic stress.

As mentioned before, the mechanical properties of the AMNF exhibit distinct tension–compression asymmetry. Namely, the Young’s modulus, yield strength, and yield strain under tension are greater than those under compression. The tension–compression asymmetry for modulus is stronger at higher temperatures, whereas that for yield strain and yield strength does not exhibit remarkable temperature dependence. It is extensively reported that the yield behaviors of MNFs are affected by the initial surface stress state [62]. Therefore, we analyze the hydrostatic stress distribution of all atoms within the atomistic model at different temperatures. The coupling effect of the initial stress state of atoms in the solid surface layers and ligament cores is of significant importance in comprehending the tension–compression asymmetry of the mechanical properties of MNFs. Figure 10a and Figure 10b depict the hydrostatic stress distribution for surface atoms and interior atoms, respectively, after relaxation at various temperatures. Figure 10c provides a comparative analysis of the hydrostatic stress distribution between surface atoms and interior atoms at 300 K. A positive hydrostatic stress implies a tensile stress state, while a negative hydrostatic stress represents a compressive stress state. As illustrated in Figure 10a, the majority of surface atoms are under tensile stress, with peak values occurring at approximately 0.047 GPa. Quantitative data for the stress distribution are summarized in Table 4. At temperatures of 100, 200, 300, 400, 500, and 600 K, the proportion of surface atoms experiencing tensile stress is approximately 92.8%, 90.8%, 89.1%, 87.4%, 85.7%, and 84.2%, respectively. In contrast, the hydrostatic stress distribution for atoms in the ligament cores exhibits a nearly symmetric balance between compressive and tensile states, with peak stress values around 0.5 GPa (Figure 10b). As detailed in Table 2, roughly half of the core atoms are under tensile stress, while the other half are under compressive stress. Figure 10d provides the local shear stress distribution of a separate ligament of the AMNF at 300 K, with atoms color-coded according to their initial hydrostatic stress. It is evident that surface layer atoms predominantly experience tensile stress (indicated by the absence of blue atoms). In contrast, the hydrostatic stress distribution for core atoms is relatively uniform. This observation visually confirms that surface atoms are under tensile stress, while interior atoms are under compressive stress. For both surface and interior atoms, the peak values in the hydrostatic stress distribution curves decrease, and the distribution range broadens as temperature increases. However, the stress values at which these peaks occur remain largely temperature independent. Consequently, the initial surface stress state does not significantly influence the tension–compression asymmetry of yield strength and yield strain. Instead, material yield behavior is highly sensitive to extreme conditions, such as the weakest ligaments and regions of the highest stress concentration. Additionally, the methods used to determine yield stress/strain and the strain range selected for calculating Young’s modulus can also affect yield stress/strain values. Therefore, the tension–compression asymmetry of yield stress/strain does not exhibit a definitive correlation with temperature.

**Figure 11:**
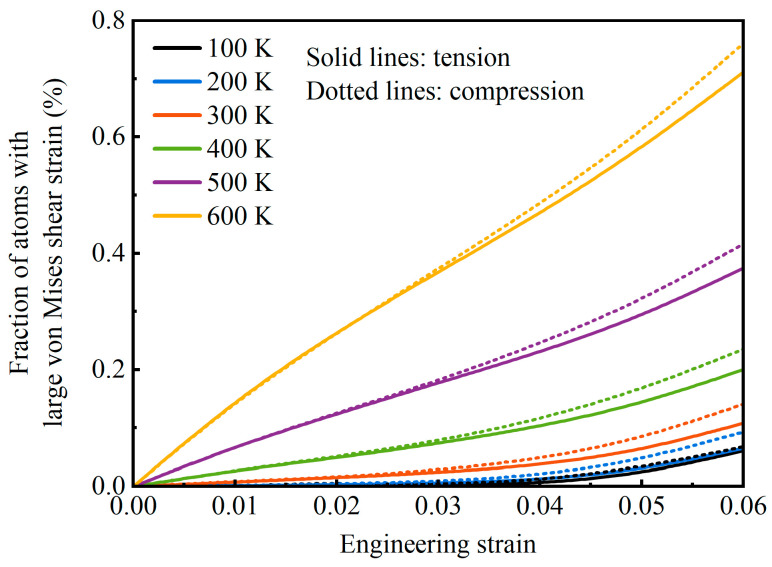
Variation of the percentage of atoms having relatively large local strain values with global strain for the AMNF under tensile loading (solid lines) and compressive loading (dotted lines) at different temperatures. The strain range of *ε* = 0−0.06 is selected to evaluate the effect of atoms having large local shear strain values on the yield behavior.

The initial surface stress state contributes to the compressive yield strength of the AMNF being lower than its tensile yield strength. Surface atoms are predominantly under tensile stress, while the ligament core atoms, particularly those near the surface layers, exhibit an average compressive stress state as a complementary response to the global stress distribution. When tensile loads are applied, the compressive stress within the core atoms counteracts the external tensile stress, thereby resisting deformation. Conversely, under compressive loading, the inherent compressive stress facilitates deformation. As a result, the compressive yield strength is reduced, while the tensile yield strength is enhanced due to this surface stress state. Figure 11 illustrates the evolution of the fraction of atoms with large von Mises shear strain as a function of engineering strain for the AMNF under tension (solid lines) and compression (dotted lines) at different temperatures. The strain range of *ε* = 0–0.06 is selected to analyze the influence of severely deformed atoms on yield behavior under both tension and compression. It is evident that, at the same temperature, the percentage of atoms in violent deformation regions is higher under compressive loading compared to tensile loading. Atomic-scale analysis reveals that regions with elevated shear strain concentrations facilitate localized deformation, reducing overall mechanical resistance and consequently resulting in the distinctive yield strength disparity between tensile and compressive loading conditions.

**Figure 12:**
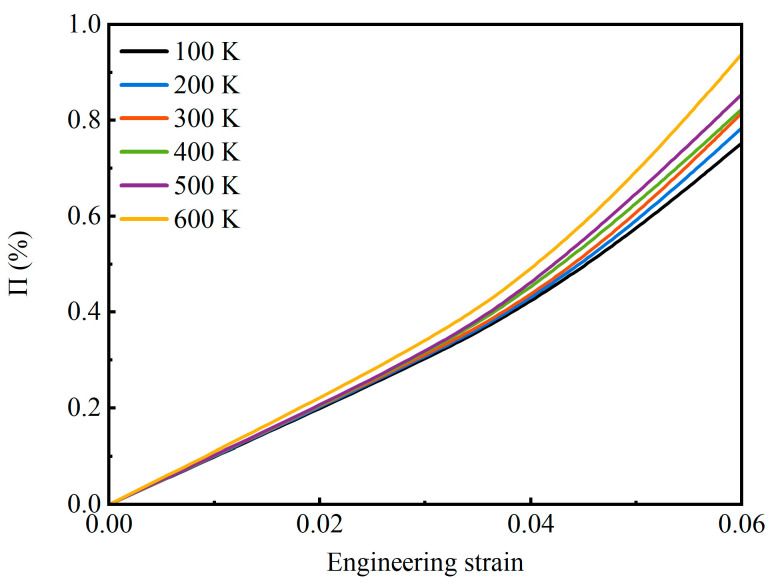
Difference in the percentage change in the surface-area-to-solid-volume ratio under tension and compression as a function of global strain at different environmental temperatures.

The Young’s modulus of the AMNF manifests strong tension–compression asymmetry. On the one hand, the tensile Young’s modulus is greater than the compressive Young’s modulus. On the other hand, the discrepancy between the tensile Young’s modulus and the compressive Young’s modulus increases with increasing temperature. This tension–compression asymmetry in Young’s modulus can be explained by the surface energy theory. Under tension loading, the specific surface area increases due to the elongation, necking, and breakage of ligaments (Figure 6a), meaning the formation of new free surfaces. These newly formed free surfaces enhance the surface energy, which is derived from external work. Hence, the stress required to reach a specified global tensile strain is enhanced. Differently, the densification under compression makes the free surface annihilate (Figure 8a), which reduces the surface energy and facilitates compressive deformation. Therefore, the stress required to reach a specified global compressive strain is reduced. This changing tendency in surface energy results in the Young’s modulus under tensile loading being greater than that under compressive loading. Figure 12 reflects the difference in the percentage change in the surface-area-to-solid-volume ratio under tension and compression as a function of global strain at various temperatures. The difference in the percentage variation in the specific surface area between under tension and compression *Π* is defined as:

*Π* = |*Π*_t_ − *Π*_c_| (3)

where *Π*_t_ and *Π*_c_ are the percentage variations in the specific surface area under tension and compression, respectively. As Figure 12 plots, as the strain increases, *Π* rises monotonically. At the same global strain, *Π* is greater for the case at higher temperatures. Therefore, as the temperatures increase, the effect of surface energy becomes more pronounced. This leads to the fact that the discrepancy between tensile modulus and compressive modulus is more intense at higher temperatures (Table 1).

## 4. Conclusions

In this study, the mechanical characteristics of CuZr amorphous metallic nanofoam (AMNF) at varying temperatures are investigated using molecular dynamics (MD) simulations. The following critical findings have been established:

(1) The key mechanical properties of the AMNF, including tensile/compressive elastic modulus, yield strength, and maximum tensile stress, show tension–compression asymmetry and strong sensitivity to temperature variations. These mechanical properties intensify with a reduction in temperature and are stronger in tension than in compression.
(2) The tension–compression asymmetry of yield strength originates from the initial surface stress state. The yield behavior is especially sensitive to localized extreme conditions in the weakest ligaments and localized regions with high stress concentrations. Additionally, the methods used to determine yield stress/strain strongly influence the calculated values. As a result, the tension–compression asymmetry in yield stress/strain is insensitive to temperature variation.
(3) The difference between tensile elastic modulus and compressive elastic modulus increases with rising temperature. This phenomenon is explained by the surface energy effect. At the same global strain, the variation in the percentage of specific surface area between tension and compression is greater at higher temperatures. Therefore, as temperatures increase, the effect of surface energy becomes increasingly prominent, leading to a greater tension–compression asymmetry of elastic modulus.
(4) Temperature variation quantitatively affects the deformation behaviors of AMNF. Although increased temperature softens the material, it also delays ligament decay and enhances the structural resistance of the porous structure under large plastic strain conditions by facilitating global homogeneous deformation. On the other hand, a higher temperature increases atomic distances and potential energy, facilitating the contact and merging of ligament surfaces into an integrated solid. This process accelerates the densification of AMNF at higher temperatures.

This research advances the fundamental knowledge of and deformation mechanisms of AMNF. The findings underscore the importance of surface energy and initial stress states in determining the material responses, offering valuable insights for optimizing nanostructured metallic glasses with pores.

## Figures and Tables

**Table 1:** Elastic modulus of the AMNF at various temperatures.

Temperature (K)	*E*_t_ (GPa)	*E*_c_ (GPa)	*E*_t_ − *E*_c_ (GPa)
100	5.863	5.704	0.158
200	5.535	5.385	0.150
300	5.189	4.960	0.229
400	4.869	4.566	0.302
500	4.499	4.176	0.322
600	4.071	3.732	0.339

**Table 2:** Yield strength and yield strain of the AMNF at various temperatures.

Temperature (K)	*ε*_yt_	*ε*_yc_	*ε*_yt_ − *ε*_yc_	*σ*_yt_ (GPa)	*σ*_yc_ (GPa)	*σ*_yt_ − *σ*_yc_ (GPa)
100	0.0430	0.0372	0.0058	0.240	0.201	0.0396
200	0.0429	0.0362	0.0067	0.226	0.184	0.0422
300	0.0422	0.0369	0.0053	0.209	0.173	0.0355
400	0.0413	0.0354	0.0059	0.191	0.153	0.0388
500	0.0402	0.0361	0.0041	0.172	0.142	0.0295
600	0.0414	0.0351	0.0063	0.160	0.124	0.0369

**Table 3:** Peak stress of the AMNF under tension at various temperatures.

Temperature (K)	*σ*_pt_ (GPa)	*σ*_pt_/*σ*_yt_ (GPa)	Strain at the Stress Drop Point	Stress at the Stress Drop Point (GPa)
100	0.277	1.15	0.153	0.196
200	0.262	1.15	0.157	0.191
300	0.242	1.15	0.182	0.168
400	0.225	1.17	0.188	0.158
500	0.206	1.20	0.208	0.142
600	0.186	1.16	0.268	0.113

**Table 4:** Percentage of atoms in initial compressive and tensile stress conditions.

Temperature (K)	Percentage of Atoms in the Initial Tensile State (%)		Percentage of Atoms in the Initial Compressive State (%)	
	Ligament Surface	Ligament Core	Ligament Surface	Ligament Core
100	92.8	47.6	7.2	52.4
200	90.8	47.8	9.2	52.2
300	89.1	48.1	10.9	51.9
400	87.4	48.3	12.6	51.7
500	85.7	48.4	14.3	51.6
600	84.2	48.6	15.8	51.4

## Data Availability

The original contributions presented in this study are included in the article. Further inquiries can be directed to the corresponding authors.
